# Benefits of immunoglobulin substitution in primary and secondary immunodeficiencies: Interim analysis of a prospective, long-term non-interventional study 

**DOI:** 10.5414/CP203952

**Published:** 2021-04-16

**Authors:** Artur Bauhofer, Sonja Schimo, Martine Klausmann

**Affiliations:** 1 Biotest AG, Dreieich, and; 2 Aschaffenburg, Germany

**Keywords:** intravenous immunoglobulin (IVIG), immunodeficiency, non-interventional study (NIS), tolerability, quality of life

## Abstract

Objective: To assess the safety, tolerability, and effectiveness of the intravenous immunoglobulin (IVIG) Intratect 100 g/L in a prospective, large-scale non-interventional study (NIS) of patients with a wide range of antibody deficiencies as well as other indications for IVIG, risk factors, and frequency of pre-treatments. Materials and methods: Patients were enrolled at 53 practices and clinics in Germany. After recording of baseline information, each patient was treated according to need, as judged by the physician and guided by the SmPC. Relevant data were acquired from medical records, and the patients completed questionnaires to assess treatment satisfaction and quality of life (QoL). Results: At cut-off for this interim analysis, 488 patients were enrolled (planned: 1,000). 47% were male, age 16 – 91 (median 61) years, with treatment durations up to 2,225 (median 282) days. Indications were primary (32%) and secondary (61%) immunodeficiencies, immune thrombocytopenia (4%), and others (3%). More than 92% of physicians recorded very good effectiveness and satisfaction. Patient satisfaction and QoL increased with time from baseline. Initially, 31% of the SID patients had inadequate IgG trough levels (< 4 g/L), including patients with (37%) and without (63%) previous IVIG treatment. Despite a relatively low IVIG dose (median 0.2 g/kg), trough levels improved: after 3 infusions, only 22% of patients had trough levels < 4 g/L, with a plateau below 17% after 6 infusions. Adverse reactions were observed at a rate of 3% per infusion, whereas 0.08% accounted for serious reactions. Conclusion: Effectiveness, safety, patient satisfaction, and QoL were good, confirming the positive benefit-risk profile of the IVIG.

**What is known about this subject**

- Primary and secondary immunodeficiencies (PID and SID) are associated with infections needing antibiotic treatment, often hospitalization, and in some cases delay of cancer treatment.
- SID is mainly induced by an underlying cancer or by the treatment of the cancer with chemotherapy, radiation, or biologicals such as rituximab.
- PID and SID patients have decreased levels of IgG and in some cases also of IgM. Substitution of immunoglobulins with intravenous immunoglobulin (IVIG) preparations aims to reduce infections.

**What this study adds**

- In this non-interventional study (NIS), IVIG treatment elevated the IVIG levels despite a relatively low drug dosage used.
- During the course of treatment, IVIG improved quality of life and treatment satisfaction of the patients.
- The IVIG was well tolerated. The need for pre-medication was substantially reduced during treatment cycles in the NIS.

## Introduction 

Intravenous immunoglobulin (IVIG) substitution therapy for symptomatic antibody deficiencies has become a well-established intervention for a wide range of primary and secondary immunodeficiency (PID and SID) conditions. In addition, IVIG has the potential to act in an immunomodulatory fashion in treating (systemic) autoimmune disorders [1, 2]. In IVIG therapy, the patient receives natural IgG antibodies prepared from pooled plasma obtained from several thousand donors [3]. 

Intratect 100 g/L is a ready-to-use, sugar-free IVIG preparation. It received first marketing authorization in 2012 and is currently available in 35 countries. Licensed indications include PID syndromes with impaired antibody production, SID with recurrent infections, ineffective antimicrobial treatment, and either proven specific antibody failure or a serum IgG level below 4 g/L. SIDs can occur as a consequence of extrinsic influences such as malnutrition, human immunodeficiency virus infection, malaria, neutropenia, hematological diseases, or as a side effect of certain medications. SIDs often have a multifactorial etiology related to both the patient’s underlying condition and its treatment, including a growing range of treatments targeting B cells. These treatments occur across a broad disease spectrum and are therefore of importance to clinicians in both primary and secondary care [4]. SID is estimated to be 30 times more common than PID, but unlike PID it is a reversible condition if the underlying cause can be resolved [5]. 

Further indications for IVIG are immune thrombocytopenia (ITP) in patients at high risk of bleeding, or before surgery, to correct platelet count; Guillain-Barré syndrome; Kawasaki disease; chronic inflammatory demyelinating polyradiculoneuropathy; and multifocal motor neuropathy [6]. The label of all IVIG preparations was recently extended on the basis of the new IVIG EU-core SmPC [7] and provides a basis for the use of IVIG replacement therapy in PID, SID, and ITP, and also in other defined neurological indications. 

This non-interventional study (NIS) was initiated once marketing authorization had been received, with the aim of confirming and further investigating the effectiveness, safety, and tolerability of IVIG substitution therapy in the prevention and treatment of infections in PID, SID, and ITP under real-life conditions, and at the same time of assessing its effect on the patients’ quality of life (QoL). A post-authorization observational study design, with a broad population of patients following up on IVIG long-term treatment, was chosen in order to overcome the limitations of controlled clinical trials and to continue intensive pharmacovigilance. 

## Materials and methods 

### Study design and conduct of the NIS 

This prospective NIS was conducted in a total at 53 out-patient and in-patient treatment centers in Germany. Before study start, ethical approval was obtained, and the relevant authorities in Germany (Paul-Ehrlich-Institute, the National Association of Physicians and the Statutory Health Insurance Association) were notified. It was planned to enroll 1,000 patients over a 5-year period. However, recruitment was slower than expected, leading to the decision to perform an interim analysis in 2019. From February 7, 2013 until July 31, 2019 (the cut-off date for this interim analysis), 488 patients were enrolled. 

### Study medication IVIG 

A single human immunoglobulin preparation for intravenous use (IVIG) was used: Intratect 100 g/L (Biotest AG, Dreieich, Germany), provided in aqueous glycine-stabilized solution containing 100 mg/mL human plasma protein; the latter comprises at least 96% IgG with a physiological distribution of subclasses (on average 57% IgG1, 37% IgG2, 3% IgG3, and 3% IgG4) [6]. 

IVIG substitution was initiated and performed at the discretion of the physician. The dosage and infusion speed varied according to indication and the individual needs of the patient, as recommended in the summary of product characteristics [6]. 

### Data acquisition 

Informed consent was obtained from the patients before documentation was started. Data were recorded at routine clinical visits. At the baseline visit, demographic and anthropometric information, diagnosis, indication for IVIG therapy, concomitant diseases, and basic treatment data were recorded. At each subsequent visit, the following data were recorded: pre-infusion plasma levels of IgG, IgM, and IgA as applicable (if routinely determined); dose and duration of IVIG infusion; and adverse events (AEs) during administration and since the previous visit, irrespective of a suspected causal relationship to the study drug. Physicians classified AEs as non-serious or serious (fatal, life-threatening, requiring hospitalization or its prolongation, persistent or significant disability/incapacity, congenital birth defect/anomaly, or other medically important conditions) and as related or not related to the IVIG treatment. AEs with a suspected relation to the IVIG were classified as adverse drug reactions (ADRs). 

At every third visit, the following additional data were documented, all in relation to the change during the period covered by the previous 3 infusions: number of infections, pre-medication used, effectiveness in terms of improvement in clinical symptoms as assessed by the physician, and tolerability of the IVIG as assessed by the physician (over 5 grades, “very good” to “not satisfactory”). Overall QoL was assessed repeatedly employing a visual analogue scale (VAS) from 0 to 15 (higher numbers indicating a better QoL) and patient satisfaction by using the TSQM (Treatment Satisfaction Questionnaire for Medication) questionnaire [8]. 

Data were primarily documented in electronic case record form (eCRFs) (~ 80%); at some sites, paper CRFs were used. The data from paper CRFs underwent single data-entry without the possibility of contacting individual sites in order to ask about inconsistencies (with the exception of queries regarding AEs). Source data validation was not performed (see below, Discussion). 

### Data management and statistical analysis 

Data management and statistical evaluation were performed by a clinical research organization independently of the sponsor. Data were analyzed by descriptive statistics: mean values, standard deviations, median values, inter-quartile ranges, and overall ranges were presented as appropriate. Categorical variables were reported by absolute and relative frequencies. SAS Version 9.4 was used to conduct the analysis. Analyses were performed, as appropriate, on the total study population and/or the full analysis set (i.e., all patients who received at least 1 IVIG infusion and for whom follow-up data after this administration were available). 

## Results 

### Patient population 

**Figure 1.**
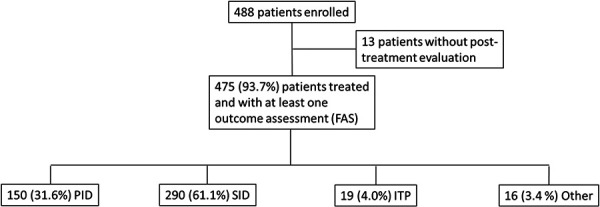
Patient enrolment and distribution. Percentages in the lowest row are based on N = 475. ITP = immune thrombocytopenia; PID = primary immunodeficiency; n.d. = not determinable; SID = secondary immunodeficiency.

At the time of the interim analysis, 488 patients had been enrolled into the NIS. Of these, 475 (94%) had received at least 1 IVIG administration and at least 1 post-infusion outcome evaluation (Figure 1). SID was the main reason for treatment (290 patients), followed by PID (150 patients). ITP was diagnosed in 19 patients, and “other” diagnoses were found in 16 patients. The latter category included patients with infection (4), undefined immunoglobulin deficiency (4), pemphigus vulgaris (2), polyneuropathy (2), Churg-Strauss syndrome (2), rheumatic disorder (1), and pancytopenia (1). 

**Figure 2.**
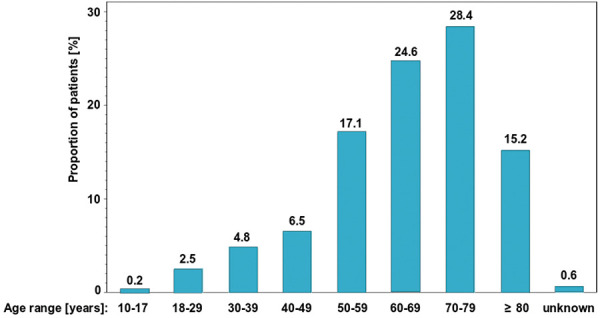
Age distribution.

The total study population analyzed included 475 patients with a mean (± SD) age of 64 (± 15) years ranging from 16 to 91 years at study enrolment (Figure 2), with only 1 patient aged below 18 years; the median age was 61 years, with an interquartile range of 52 – 71 years. Female patients accounted for 53%. Patients documented in this NIS were from all regions in Germany, and overall, they represent a typical Caucasian population. 

### Disease history and previous treatment 

The primary cause for IVIG treatment was malignancy in 279 (59%) of the 475 patients treated, followed by infectious diseases in 26 patients (6%), and autoimmune diseases in 29 patients (6%). The main indications for treatment were SID (N = 290; 61%) and PID (N = 150; 32%). The median time since diagnosis, defined as the interval between date of first diagnosis and the baseline visit, was 4.6 years, with a range of 0 – 33 years. 

The most common concomitant diseases were hypertension or vascular disease (in 59% of the 252 patients with available data) and diabetes mellitus (24%). 

The most frequent therapy encountered at baseline, i.e., before NIS start, was treatment with immunoglobulins in the PID and SID group (38% each) and corticosteroids in the ITP group (68%), whereas immunosuppressants were most frequently administered in the SID group (37%). 

### IVIG therapy 

Of the 236 patients with documented data on previous treatment, 162 (69%) received Intratect 100 g/L therapy for the first time; among these, 96 patients (41%) were treated with immunoglobulins for the first time in their life in this NIS. 

In total, the study patients received 9,724 IVIG administrations. The median treatment duration was 282 days, ranging from 1 to 2,225 days (up to 6 years). A median of 9 IVIG applications were administered per patient; overall, 254 patients (53%) received 1 – 10 infusions, 68 (14%) 11 – 20 infusions, 89 (19%) 21 – 50 infusions, and 64 (13%) > 50 infusions. 

### PID and SID treatment 

The overall number of infusions was roughly the same in the PID and SID groups. In total, 440 patients (PID 150, SID 290) received 9,358 IVIG infusions (PID 4,449, SID 4,909), median numbers of 18 (PID) or 9 (SID) administrations per patient were recorded (Table 1). The mean observation period for PID and SID patients was 23 and 16 months, and the median single doses were 10 g and 18 g (0.2 g/kg body weight for both indications, corresponding to 12.3 g per patient; the mean value in these indications was also 0.2 g/kg). Some patients received up to 80 g. The median treatment interval was 29 days for PID and 30 days for SID patients. On the basis of the total study population, PID and SID patients received annually a median of 15 and 14 infusions, respectively. The median initial speed of infusion was 0.5 mL/kg/h, and the median greatest infusion speed was 1.6 mL/kg/h. The maximum infusion speed used was 7 mL/kg/h. 

The mean trough IgG levels on IVIG therapy were similar between the IgG-pretreated (40%) and IgG-naïve (60%) patients at treatment start and were lowest before entry into the NIS. They subsequently increased following the first infusions of the study IVIG. In the PID group, they increased from a median of 6.66 g/L before infusion 1 (N = 97) to 7.63 g/L before infusion 6 (N = 63) and 7.13 g/L before infusion 12 (N = 43). In the SID group, they increased from a median of 5.25 g/L before infusion 1 (N = 196) to 6.97 g/L and 7.07 g/L prior to infusion 6 (N = 117) and 12 (N = 82), respectively. 

Closer inspection of the SID group reveals that IgG levels increased more in previously untreated patients (N = 166). The median IgG level rose from 4.78 g/L (N = 121) at baseline to 6.63 g/L after 3 infusions (N = 101) and to 6.85 g/L after 6 (N = 95) infusions. No increase in the median levels was observed for IgA and IgM during the same period. 

**Figure 3.**
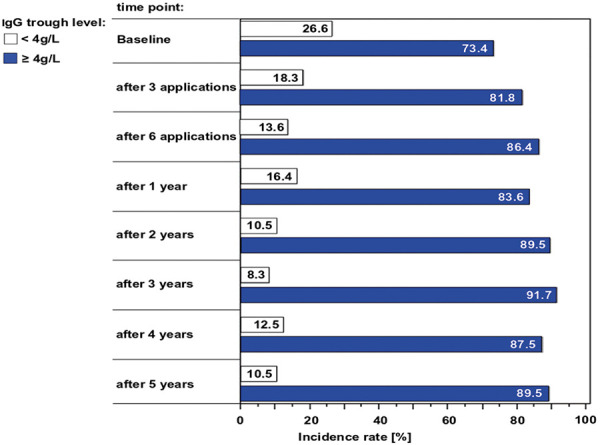
Proportion of patients with IgG trough level < 4 g/L and ≥ 4 g/L over time.

The proportion of patients with trough levels below the 4 g/L threshold decreased throughout the observation period (from 27% at baseline to 10.5% after 5 years (Figure 3)). 

### ITP treatment 

All patients in the ITP group received less than 10 infusions, with a median of 4 IVIG applications and a treatment duration of 8 days (Table 1). The median treatment interval between infusions was 2 days. Patients received a median dose of 0.4 g/kg body weight (corresponding to 30 g for a typical 75-kg patient). The median initial speed of infusion was 1.2, and the median greatest infusion speed was 2.3 mL/kg/h. 

### Use of pre-medication at baseline and after 1 year 

**Figure 4.**
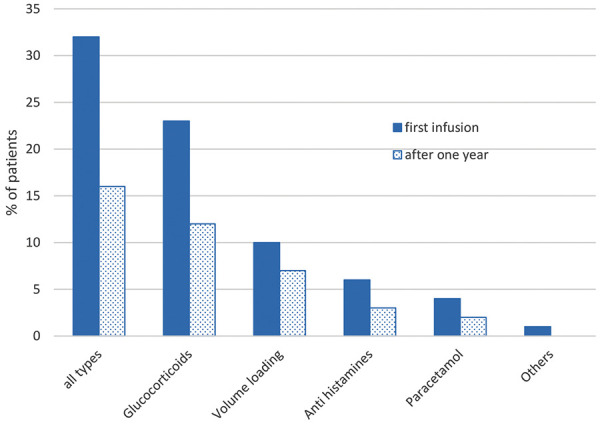
Pre-medication provided before intravenous immunoglobulin infusion. Multiple mentioning is possible (some patients received more than 1 pre-medication).

Before the first IVIG application in the NIS, 32% of the patients received pre-medication. After 1 year of IVIG treatment, this fraction was halved to 16% (Figure 4) (Table 2). The pre-medications most frequently used were glucocorticoids (in 23% of the patients at study start and 12% after 12 infusions). Other commonly used pre-medications were volume loading with saline, antihistamines, and paracetamol. SID patients required more pre-medication than did other patient groups (pre-medication at baseline and after 12 IVIG 100 g/L infusions: SID, 38%/20.0%; ITP, 32%/–, PID, 22%/10%; other, 13%/13%). 

### Infections 

39% of the patients reported at least 1 infection during the 3 months before study entry (Table 3). In the SID subgroup, 107/231 (46%) had had at least 1 infection in this period, and about half of them (21%) had had 2 or more infections. Not only was the infection rate highest in the SID group, but antibiotic use, the severity of the most recent infection, and hospitalization were most pronounced in this group. Overall, 9% of patients had experienced at least 1 severe bacterial infection, requiring hospitalization, during this period. Most of these occurred in the SID group (12% of patients). 

The mean annual infection rate during study participation was 2.7, compared with 2.6 in the 3 months preceding study entry (Table 3), not differentiating between patients who had or had not previously been treated with IVIG – this comparison varied notably between patient groups. In the SID group, of the 290 patients, 129 (45%) developed at least 1 infection, and 90 (31%) patients developed an infection needing antibiotic treatment (Table 4). Interestingly, only 34 of 129 patients (26%) received an IVIG dose of at least 0.35 g/kg body weight. For patients with infections requiring antibiotic treatment, even fewer patients (19; 21%) were dosed with 0.35 g/kg body weight or more. 

On IVIG therapy, the overall number and severity of infections was similar between IgG-pretreated (N = 158, 40%) and IgG-naïve (N = 238, 60%) patients. Mean severe bacterial infections requiring hospitalization were slightly more frequent in the SID than in the PID group. Median annual rates of severe bacterial infections requiring hospitalization were zero in all groups. 

The most frequently documented infections were: bronchitis (25 events, 45%), nasopharyngitis (24 events, 5%), pneumonia (17 events, 4%), undefined infections (7 events, 1.5%), urinary tract infections (6 events, 1.3%). 

### Quality of life, determined by VAS (0 – 15) 

At baseline, 183 patients responded to the QoL questionnaire. The overall mean ± SD (median) score was 9.8 ± 3.3 (10) at baseline, improving to 10.7 ± 3.0 (11) after 3 infusions and to 11.0 ± 2.9 (12) after 1 year. Changes from baseline (mean ± SD) were: overall, 0.9 ± 2.3 after 3 infusions and 0.6 ± 3.0 after 1 year; in the PID subgroup, these were 0.8 ± 1.9 after 3 infusions and 0.4 ± 2.4 after 1 year; in the SID subgroup, these were 1.0 ± 2.5 after 3 infusions and 0.8 ± 3.2 after 1 year. Median values are displayed in Table 5. Overall, these results indicate a continuous improvement in QoL. 

### Patient satisfaction determined with the TSQM 

The TSQM has 4 domains: “effectiveness”, “side effects”, “convenience”, and “global satisfaction” (Table 5). Over time (baseline, 3 months, and 1 year), satisfaction increased for “all patients” and also for most of the disease groups. For “effectiveness”, an improvement from baseline to 1 year of 6.5, from 72.7 to 79.2 points in the median was observed. No change for “side effects” could be observed, as at all time-points the highest possible value of 100 points was obtained. “Convenience” of the study medication improved by 8.4, from 69.4 to 77.8 points. The highest improvement, of 16.6, was observed for “global satisfaction”, from 66.7 to 83.3 points. 

### Adverse events and suspected adverse drug reactions 

During the course of the study, 198/475 patients (42%) experienced 923 AEs (Table 6). In the SID and ‘other’ group, a larger proportion of patients had AEs (47% and 44%, respectively) than patients in the PID and ITP groups (33% and 21%). 60 patients (13%) experienced a total of 212 serious AEs. Five patients (1.1%) died (4 with SID and 1 with PID); none of the deaths were related to an IVIG infusion, and all were related to underlying diseases (i.e., myocarditis, disease progression, sepsis, suicide, and pancreatic cancer). 

Events of special interest were predefined; among these, thromboembolic events occurred in 4 patients, 2 each in the PID and the SID group. In the PID group, these were “cerebrovascular accident” and “pulmonary embolism”, and in the SID group, they were “deep vein thrombosis” and “thrombosis”. These events were not considered to be associated with the use of the study medication, because the time to onset made this implausible and/or because other risk factors in the patients’ medical history presented alternative explanations. 

46 patients (10%) had at least 1 hypersensitivity reaction (allergic up to anaphylactic reactions or non-allergic infusion-related reactions); 37 of those patients were in the SID group. The most frequent event overall was ‘infusion-related reaction’ (27 patients, 6%), experienced by 21 patients (8%) in the SID group and 6 (4%) in the PID group. Further events in the SID group were hypersensitivity (5 patients, 1.7%), rash (3 patients, 1.0%), pustular rash (2 patients, 0.7%), vomiting, chills, and face swelling (each 1 patient, 0.3%). 

Hemolysis occurred in only 1 patient in the “other” group (hemolytic anemia); this was not considered related to the IVIG administration because of the implausible temporal relationship (1 month after the last application). No events of special interest were recorded in the following areas: aseptic meningitis; renal failure; suspected transmission of viral pathogen infection; transfusion-related acute lung injury; or systemic lupus erythematosus. 

The incidence of AEs per infusion (irrespective of causality) was 9% (923/9,724) for all patients: 3% (150/4,449) for PID, 15% (735/4,909) for SID, 6% (5/87) for ITP, and 12% (33/279) for “other” patients. 

In 139/475 patients (29%) a suspected relationship or association with the IVIG was recorded for 299 events (therefore classified as ADRs); 4 patients (0.8%) experienced 8 serious ADRs. This corresponds to a per-infusion ADR frequency of 3% (299/9,724) for all patients, 1.6% (71/4,449) for the PID patients, and 4% (219/4,909) for the SID patients. The frequency of serious ADRs was 0.08% for the total population, 0.09% in the PID, and 0.08% in the SID group. 

Therapeutic measures to treat AEs were taken for 166 (35%) of patients; these included discontinuation of infusion (13%) and changes in concomitant medication (9%), but the most frequent action to treat AEs was recorded as “other” (27%). 

The most common preferred term recorded was “chills” (58/475 patients, 12%; Table 7). The following AEs occurred in at least 5% of the patients: infusion-related reactions (28 patients, 5.9%), nasopharyngitis and nausea (24 patients, 5% for each term). 

Over all applications, physicians rated the tolerability of the IVIG as “good” for 53% or “very good” for 43%, together “good” and “very good” for 96%, as “moderate” for 3.5%, and as “not satisfactory” for only 0.7%. Effectiveness was rated similarly by the physicians as “good” or “very good” for 92% of the patients, as “moderate” for 7%, and as “not satisfactory” for only 0.7%. 

## Discussion 

The patient demographics (age and sex) in this interim analysis corresponded to that expected in immunodeficiency indications. The proportion of patients with SID was about twice as high as that of PID patients, and other indications including ITP were only weakly represented. Thus, for data analysis, the main focus was on the PID and SID groups. It is to be noted that most of the study patients were elderly (median age 61 years); therefore, the findings of this study may be considered representative for adults and elderly patients. 19% of the patients treated received study treatment for ≥ 1 year and 13% for ≥ 2 years, so that this analysis, despite its interim nature, conveys meaningful medium- to long-term information. 

The physicians expressed overall satisfaction with the effectiveness of the treatment. Objectively, the proportion of patients with an IgG deficiency (in this analysis the minimum level was taken to be 4 g/L) decreased after the commencement of IVIG administration: at baseline it was 27%, after 1 year it had decreased to 16%, and after 5 years (though only a small number of study patients had so far received such long-term treatment) it had decreased to 10.5%. Moreover, pre-medication could be reduced by ~ 50% during the course of the study. The most common pre-medications were glucocorticoids, followed by volume loading (isotonic NaCl, Ringer’s solution). The effectiveness and safety of the IVIG did not appear to differ largely between patients with different treatment indications, e.g., PID, SID, ITP, and “other indications”. The AE frequency was higher among SID patients (15%) than among PID patients (3%). This higher frequency may be explained by the higher age in the SID group (mean 70 years, compared with 61 years in the PID group) and the underlying cancer with its immune-suppressive treatment (irradiation, chemotherapy, and biologicals). However, this conclusion is less firm for the ITP and “other” groups, as the numbers of patients in these were much smaller than in the PID and SID groups. 

Most important for assessing the effectiveness of any treatment of immune deficiency is the infection rate before and after start of IVIG therapy [9]. In the present study, the overall annual infection rate was 2.6 before and 2.7 after treatment, i.e., almost unchanged. Similar infection rates have been found in comparable studies. For PID an annual infection rate of 3.3 under IVIG treatment was determined in a meta-analysis [10]. However, the baseline value relies at least partly on the patients’ recollection and is therefore somewhat unreliable. A striking observation is that a considerable proportion of the patients in the SID group who experienced an infection (95; 74%) or had a severe infection requiring antibiotics (71; 79%) were treated at levels below 0.4 g/kg body weight. In the median, patients were treated with 0.2 g/kg body weight. According to the EMA guidance, the recommended dosage for SID is between 0.2 and 0.4 g/kg [7], and the individual dosage should be adjusted as necessary to achieve optimum protection against infection. Therapy is guided mainly by the clinical course, taking into consideration the individual IgG trough levels in conjunction with the incidence of infection. Some guidelines [11] and publications [12] argue that treatment should be initiated with at least 0.4 g/kg and that the dose should then be adjusted according to the patient’s treatment needs. A recent study by Weide et al. [13] showed that SID patients with IgG trough levels (7 g/L or more) had a significantly reduced incidence of all types of infections as well as less severe infections requiring antibiotic treatment, compared to patients with lower IgG levels (< 7 g/L). Furthermore, a meta-analysis [9] provided evidence that in SID patients the risk of pneumonia can be progressively reduced by higher trough IgG levels of up to at least 10 g/L, with a median IVIG dose administered of 0.5 g/kg body weight every 4 weeks. Overall, these results support the contention that patients with symptomatic antibody deficiency benefit from a targeted IgG level-adjusted therapy. 

Most of the SID patients (69%) observed in this study had already been treated with various drugs before entering the study. These factors might have contributed to a substantial scatter of data, as is already seen in the difference between the PID and the SID patient groups (Table 3). The number of infections and infections requiring hospitalization were low both for the total study population and, especially, in the SID patient group [10]. 

Both the patients’ overall QoL and their satisfaction with the treatment increased during the course of the NIS. The TSQM is a well-established questionnaire [8]. Tolerability was found to be excellent. In particular, there were few ADRs (3% for all infusions) and very few serious ADRs (0.08%). The ADR rates were in a range comparable to that found with other IVIG preparations [10]. 

Thus, all in all, patients benefited from the treatment with IVIG, especially (as mentioned above) the elderly patient cohort investigated. 

No new safety relevant information regarding the IVIG Intratect 100 g/L was revealed in this NIS [6]. Similar effectiveness and safety data were obtained in an earlier NIS in which the IVIG Intratect 50 g/L was studied, also in PID and SID [14]. 

Limitations of this study, apart from the interim nature of the present analysis, lie principally in the inherently open nature of an NIS and the limited degree of monitoring and data-cleaning that can be practiced within its framework (see Data Acquisition for a summary of the methods used). Thus, data discrepancies cannot be ruled out completely. Since this study is an observational study, epidemiological methods were employed for data analyses, and all data acquired could only be analyzed descriptively. 

## Conclusion 

The physicians’ assessment of effectiveness and the patients’ satisfaction with the treatment and their quality of life were good. High values were obtained during the study, and in several cases, they improved in comparison with baseline.

- Therapy with Intratect 100 g/L increased IgG mean trough levels from 4.78 g/L at baseline to 6.85 g/L after 6 infusions.
- The mean IVIG dose, 0.2 g/kg body weight, was low compared with other studies, especially for SID patients.
- The numbers of infections, and of infections requiring hospitalization, were low, both in the total study population and especially in the SID group.
- After 1 year of IVIG therapy, the number of patients requiring pre-medication was reduced by 50%.
- The IVIG was well tolerated. Our data confirm the good safety and tolerability profile and thus the favorable benefit-risk profile of this IVIG in these indications.

## Acknowledgment 

We thank Paul Woolley for assistance with writing the manuscript. 

## Authors’ contribution 

Artur Bauhofer: Designing the study, supervision of the study, analysis of the study, interpretation of the study results, main author of the publication 

Sonja Schimo: Analysis of the study, interpretation of the study results 

Martine Klausmann: Clinical study coordinator, analysis of the study, interpretation of the study results. 

## Funding 

The study was funded by Biotest AG, Dreieich, Germany. 

## Conflict of interest 

A. Bauhofer and S. Schimo are employees of Biotest AG. 

**Table 1.** Treatments.

Therapy	PID (N = 150)	SID (N = 290)	ITP (N = 19)	Other (N = 16)	Total (N = 475)
Treatments before NIS start					
Immunoglobulins	57 (38.0%)	110 (37.9%)	3 (15.8%)	8 (50.0%)	178 (37.5%)
Immunosuppressants	7 (4.7%)	106 (36.6%)	1 (5.3%)	3 (18.8%)	117 (24.6%)
Corticosteroids	5 (3.3%)	18 (6.2%)	13 (68.4%)	5 (31.3%)	41 (8.6%)
Other	3 (2.0%)	31 (10.7%)	2 (10.5%)	4 (25.0%)	40 (8.4%)
None	84 (56.0%)	91 (31.4%)	4 (21.1%)	5 (31.3%)	184 (38.7%)
IVIG treatment (median)					
Treatment duration [days]	415.0 [129 – 1135]	269.5 [85 – 743]	8.0 [4 – 17]	431.5 [50 – 678]	282.0 [84 – 877]
Number of applications per subject	18.0 [6 – 49]	9.0 [3 – 25]	4.0 [3 – 6]	10.0 [3 – 22]	9.0 [4 – 29]
Annual number of applications	14.8 [12 – 25]	13.7 [11 – 19]	n.c.	16.1 [12 – 23]	14.5 [12 – 23]
Dose per kg body weight [g/kg]	0.2 [0.1 – 0.2]	0.2 [0.1 – 0.3]	0.4 [0.3 – 0.5]	0.2 [0.2 – 0.5]	0.2 [0.1 – 0.3]
Speed of infusion [mL/kg/h]	0.9 [0.6 – 2.4]	1.1 [0.6 – 1.9]	1.5 [1.1 – 1.9]	1.2 [0.9 – 2.0]	1.1 [0.7 – 2.0]
Initial speed of infusion [mL/kg/h]	0.7 [0.3 – 0.9]	0.5 [0.3 – 0.8]	1.2 [0.5 – 1.7]	1.2 [0.2 – 1.4]	0.5 [0.3 – 0.9]
Maximal speed of infusion [mL/kg/h]	1.8 [0.8 – 1.9]	1.4 [1.0 – 2.0]	2.3 [1.7 – 3.3]	1.9 [0.8 – 5.6]	1.6 [1.0 – 2.1]

Interquartile ranges are shown in square brackets. IVIG = intravenous immunoglobulin; ITP = immune thrombocytopenia; PID = primary immunodeficiency; n.c. = not calculated because of the small number of patients; SID = secondary immunodeficiency.

**Table 2.** Pre-medication before first IVIG application and after 12 IVIG applications in the study.

Pre-medication	PID (N = 150)	SID (N = 290)	ITP (N = 19)	Other (N = 16)	Total (N = 475)
Before to first IVIG infusion in the NIS					
Number of patients with pre-medication	33 (22.0%)	111 (38.3%)	6 (31.6%)	2 (12.5%)	152 (32.0%)
Glucocorticoids	22 (14.7%)	81 (27.9%)	4 (21.1%)	2 (12.5%)	109 (22.9%)
Volume loading	10 (6.7%)	33 (11.4%)	3 (15.8%)	0	46 (9.7%)
Antihistamines	8 (5.3%)	17 (5.9%)	0	1 (6.3%)0	26 (5.5%)
Paracetamol	3 (2.0%)	16 (5.5%)	1 (5.3%)	0	20 (4.2%)
After 12 IVIG infusions					
Number of patients with pre-medication	15 (10.0%)	58 (20.0%)	0	2 (12.5%)	75 (15.8%)
Glucocorticoids	12 (8.0%)	44 (15.2%)	0	1 (6.3%)	57 (12.0%)
Volume loading	5 (3.3%)	25 (8.6%)	0	1 (6.3%)	31 (6.5%)
Antihistamines	4 (2.7%)	8 (2.8%)	0	0 (0.0%)	12 (2.5%)
Paracetamol	1 (0.7%)	6 (2.1%)	0	0 (0.0%)	7 (1.5%)

Multiple mentioning is possible (some patients received more than 1 pre-medication). IVIG = intravenous immunoglobulin; ITP = immune thrombocytopenia; PID = primary immunodeficiency; SID = secondary immunodeficiency.

**Table 3.** Infection episodes in the last 3 months before study entry and in 1 year of study participation.

Infections before study entry					
	PID (N = 105/150)	SID (N = 231/290)	ITP (N = 14/19)	Other (N = 12/16)	Total (N = 362/475)
Number of infected patients in the last 3 months N (%)*	26 (25%)	107 (46%)	2 (14%)	3 (25%)	138 (38%)
Annual infection rate (mean)	1.6	3.3	n.d.	1.0	2.6
Infections during study participation					
Annual infection rate (mean)	3.3	2.6	0	0.7	2.7
Annual rate of AB-requiring infections^#^ (mean)	3.0	0.7	0	0.31	1.4
Annual rate of infections with hospitalization (mean)	0.01	0.06	0	0.19	0.05

^#^Including antibiotic prophylaxis. In each column N shows the number of patients in the group and the number of these for whom data were available. *The numbers for the last 3 months before study participation relies on the patients’ recollection. The annual infection rate was calculated on the basis of 3 months of values. AB = antibiotics; ITP = immune thrombocytopenia; PID = primary immunodeficiency; n.d. = not determinable; SID = secondary immunodeficiency.

**Table 4.** Infections in SID patients and IVIG dosing (N = 290 with SID).

	Infected patients	Infections	Antibiotic treated pat.	Antibiotic treatments	Hospitalized patients	Hospitalization
SID	129 (45%)	347	90 (31%)	193	6	9
SID treated with ≥ 0.35 g/kg	34 (26%)	n.d.	19 (21%)	n.d.	0	n.d.

n.d.= not determinable; SID = secondary immunodeficiency.

**Table 5.** Quality of life (QoL, median values).

	PID (N = 150)	SID (N = 290)	ITP (N = 19)	Other (N = 16)	Total (N = 475)
General QoL determined with VAS (score 0 – 15)					
Baseline	11.0	10.0	11.0	7.0	10.0
After 3 infusions	12.0	11.0	12.0	10.0	11.0
After 1 year	12.0	11.0	n.d.	10.0	12.0
TSQM: effectiveness (0 – 100 points)					
Baseline	75.0	66.7	66.7	62.5	72.7
After 3 infusions	83.3	66.7	33.3	75.0	70.7
After 1 year	83.3	75.0	n.d.	87.7	79.2
TSQM: side effects (0 – 100 points)					
Baseline	100	100	100	58.8	100
After 3 infusions	100	100	100	95.8	100
After 1 year	100	100	n.d.	91.7	100
TSQM: convenience (0 –100 points)					
Baseline	83.3	66.7	72.2	69.4	69.4
After 3 infusions	88.9	72.2	76.4	86.1	77.8
After 1 year	94.4	72.2	n.d	80.6	77.8
TSQM: global satisfaction (0 – 100 points)					
Baseline	83.3	66.7	70.8	66.7	66.7
After 3 infusions	83.3	75.0	33.3	91.7	75.0
After 1 year	83.3	83.3	n.d.	83.3	83.3

ITP = immune thrombocytopenia; PID = primary immunodeficiency; n.d. = not determinable; SID = secondary immunodeficiency.

**Table 6.** Adverse events and suspected adverse drug reactions.

	PID (N = 150)	SID (N = 290)	ITP (N = 19)	Other (N = 16)	Total (N = 475)
Adverse events (AE)	50 (33%) 150	137 (47%) 735	4 (21%) 5	7 (44%) 33	198 (42%) 923
Mild	26 (17%) 49	70 (24%) 226	1 (5%) 1	5 (31%) 8	102 (21%) 284*
Moderate	22 (15%) 55	93 (32%) 327	1 (5%) 1	3 (19%) 8	119 (25%) 391*
Severe	13 (9%) 26	36 (12%) 134	1 (5%) 1	4 (25%) 11	54 (11%) 172*
Adverse drug reactions (ADR)	37 (25%) 71	95 (33%) 219	3 (16%) 3	4 (25%) 6	139 (29%) 299
Serious AE	15 (10%) 37	42 (14%) 158	0	3 (19%) 17	60 (13%) 212
Serious ADR	2 (1%) 4	2 (1%) 4	0	0	4 (1%) 8
Fatal AE	1 (1%) 1	4 (1%) 5	0	0	5 (1%) 6

Above, number (%) of patients; below, number of events. ITP = immune thrombocytopenia; PID = primary immunodeficiency; SID = secondary immunodeficiency.

**Table 7.** Adverse events and adverse drug reactions by MedDRA preferred term occurring in at least 5% of all patients treated.

Preferred term	Adverse events (AE)	Adverse drug reactions (ADR)
Chills	58 (12%)	52 (11%)
Infusion-related reaction	28 (6%)	28 (6%)
Nasopharyngitis	24 (5%)	0
Nausea	24 (5%)	0

Adverse events (AE) include all events observed without assessment of a causal relationship with the administered IVIG. In contrast, adverse drug reactions (ADR) have a possible relation to the IVIG application. MedDRA = Medical dictionary for regulatory activities.
